# ﻿*Acorusshannai* (Acoraceae), a new species from Southern China

**DOI:** 10.3897/phytokeys.251.139141

**Published:** 2025-01-20

**Authors:** Zhuo Cheng, Xinyu Liu, Nianting Yu, Weizhe Chen, Haofeng Zhao, Feifei Li, Chunlin Long

**Affiliations:** 1 Key Laboratory of Ecology and Environment in Minority Areas (Minzu University of China), National Ethnic Affairs Commission of China, Beijing 100081, China; 2 College of Life and Environmental Sciences, Minzu University of China, Beijing 100081, China; 3 China National Botanical Garden (North Garden), Beijing 100081, China; 4 Institute of National Security Studies, Minzu University of China, Beijing 100081, China; 5 Key Laboratory of Ethnomedicine, Ministry of Education of China (Minzu University of China), Beijing 100081, China

**Keywords:** *Acorusgramineus> var. macrospadiceus*, *Acorusmacrospadiceus*, Guizhou Province, new taxon, taxonomy

## Abstract

*Acorusshannai* (Acoraceae) is a commonly used seasoning in southern China. It was previously misidentified as *A.macrospadiceus* (Yamam.) F. N. Wei & Y. K. Li before. Through comparison of morphological characteristics, distribution locations, and type specimen, we determined that the elevation of A.gramineusvar.macrospadiceus Yamam to species status was incorrect. Therefore, we propose a formal description of a new species following nomenclature regulations. Based on morphological and plastid genomic data, this study formally describes and illustrates *Acorusshannai*, distributed in the Qiandongnan area of Guizhou Province and its surroundings, confirming it as a new species within the genus *Acorus*. This species is morphologically and phylogenetically distinct from other members of the *A.gramineus* group. Key distinguishing features include a strong fennel odor (*vs.* aromatic in “*A.tatarinowii*” and *A.gramineus*), fruit with prominent dorsal sutures (*vs.* inconspicuous dorsal sutures in “*A.tatarinowii*” and *A.gramineus*), and a leaf-shaped spathe that is about 2–3 times as long as the spadix (*vs.* more than 3 times in “*A.tatarinowii*”). Phylogenetic analysis showed that *A.shannai* is closely related to the other species in the *A.gramineus* group. The recognition of *A.shannai* is not only important for the species diversity and phylogenetic relationship of *Acorus*, but also can avoid the drug safety caused by using other *Acorus* species as *A.shannai* to eat and promote the conservation of *A.shannai* resources.

## ﻿Introduction

*Acorus* L., a helophyte and rheophyte genus, occupies a unique phylogenetic position as one of the most isolated genera among angiosperms. As a basal group of monocots, it forms its own family and order (Sokoloff et al. 2024). Species within *Acorus* hold multiple values, including cultural, medicinal, nutritional, ornamental, economic, and ecological uses (Cheng et al. 2020). Notably, *Acoruscalamus* L. has been a symbolic plant in Chinese culture for centuries and is used as a ceremonial object during festivals throughout the country (Shu et al. 2018a). Additionally, *Acorusmacrospadiceus* (Yamam.) F. N. Wei & Y. K. Li and *A.gramineus* Sol. ex Aiton have been used by different linguistic groups as spices to enhance the flavor of meat (Shu et al. 2018b; Sun et al. 2024). All species of *Acorus* possess medicinal value, especially “*A.tatarinowii*”, which has been used as herbal medicine in China for more than 2000 years. Since its first inclusion in the Chinese Pharmacopoeia, it has served as the foundational plant for traditional Chinese medicine known as Shichangpu, used for various medicinal purposes, especially in treating central nervous system diseases (Wang et al. 2014).

Despite the multiple values of *Acorus* species, their taxonomy remains unclear (Cheng et al. 2020; Sokoloff et al. 2024). The early phase of taxonomic studies of *Acorus*, which was then classified in Araceae family, culminated in an important monograph by H.W. Schott published in 1860. This work recognized as many as nine species within the genus, primarily based on diagnostic characteristics such as size and shape of key organs, like leaves, inflorescence (spadices), and spathe, as well as the ratio of spathe to spadix length. In a subsequent fundamental monograph on Araceae published in 1905, A. Engler recognized only two species in the genus, *A.calamus* and *A.gramineus* (Sokoloff et al. 2023b). Although some later authors recognized more than two species within the genus, Engler’s idea of two major groups of *Acorus* has remained widely accepted.

Currently, the genus *Acorus* is typically divided into two taxonomic groups recognized at the species level: the *A.calamus* group and the *A.gramineus* group. The *A.calamus* group has a wide native range that includes temperate North America and some parts of temperate to mountainous tropical Asia, extending northward into Russia. Taxonomically, this group consists of three closely related and morphologically similar species: diploids (*A.americanus*), triploids (*A.calamus*) and tetraploids (*A.verus*). This classification is supported by phylogenic and morphological evidence (Sokoloff et al. 2024a). The nomenclature for species in this group has been established through several papers: on *A.verus* (Sokoloff et al. 2023c), on “*A.tatarinowii*” (Sokoloff et al. 2023a), on the other species described by Schott (Sokoloff et al. 2023b), and on the typification and conservation of *A.americanus* (Sokoloff and Sennikov 2023). Research indicates that the origin of the triploid taxon is in eastern Kazakhstan (Sokoloff et al. 2024b). The *A.gramineus* group is native to East and Southeast Asia (Li 1979; Li et al. 2010). Among the most comprehensive literature sources on Southeast Asia plants – such as “Flora of China”, “Flora Malesiana”, and “Flora of Thailand” —Engler’s classification is followed by accepting one polymorphic species within the *A.gramineus* group. However, some other authorities, particularly in China, recognize multiple species within this group (Li 1979; Cheng et al. 2020).

China has one of the most extensive distributions of the *A.gramineus* group. Previous research identified three species within this group through DNA barcoding and chemical composition: *Acorusmacrospadiceus*, “*A.tatarinowii*” and *A.gramineus* (Cheng et al. 2020). However, recent studies have confirmed that “*A.tatarinowii*” does not belong to the *A.*gramineus group but represents a synonym of the *A.calamus* group (Sokoloff et al. 2023a). This indicates that “*A.tatarinowii*” lacks a valid designation and requires redescription. The primary purpose of this study is to solve the nomenclatural issue surrounding *A.macrospadiceus*, with a more thorough description of *A.tatarinowii* to be conducted separately in the future.

*Acorusmacrospadiceus* (Yamam.) F.N. Wei & Y.K. Li was described as a new taxon by Wei and Li (Wei and Li 1985). However, the name has not been properly published according to botanical nomenclature standards. Wei and Li intended to create a new combination based on “A.gramineusvar.macrospadiceus Yamam.”, which was proposed by Yamamoto (type specimen collected from Hainan Province) in 1943 (Yamamoto 1943) but lacked a validating Latin description, requirements that were strictly enforced from 1935–2011. Although Wei and Li 1985 (Wei and Li 1985) provided a Latin description for their species, they failed to designate a type specimen as mandated for new species publication since 1958. After studying the type specimen of Acorusgramineusvar.macrospadiceus, along with the morphology and chloroplast genome of Acorus, we concluded that “*Acorusmacrospadiceus*” described by Wei & Li from southwest China is indeed a distinct species. Yet, it is not the same as Acorusgramineusvar.macrospadiceus. Based on our careful examination of the type specimen, we determined that Wei and Li incorrectly elevated Acorusgramineusvar.macrospadiceus to species status. Therefore, it is necessary to publish a new taxon and designate a type specimen for the correct *Acorusmacrospadiceus*.

## ﻿Material and methods

### ﻿Morphology

This study was based on field observations and detailed examinations of herbarium specimens. Specimens were collected from Leishan County, Qiandongnan Miao and Dong Autonomous Prefecture during field expeditions in August 2021. Herbarium specimens collected from northwest Yunnan were deposited at the Herbarium, Kunming Institute of Botany, Chinese Academy of Sciences (**KUN**) (Thiers 2020). A comparative study of herbarium collections at Chinese Academy of Sciences (**PE**), Kunming Institute of Botany, Chinese Academy of Sciences (**KUN**), Guangxi Institute of Botany (**IBK**), South China Botanical Garden, Chinese Academy of Sciences (**IBSC**), College of Biology and Environmental Sciences, Jishou University (**JIU**), and Guangxi Institute of Chinese Medicine & Pharmaceutical Science (**GXMI**) revealed an undescribed taxon in the genus *Acorus*. Dried specimens were examined using a dissecting microscope (XTL-Iab, Beijing Keyi Electro-optical Instrument Factory). Detailed observations and measurements of the collected individuals included the rhizoid, fibrous roots, leaf, petiole leaf, bract, flower, and fruit. The conservation status was assessed using the IUCN Red List categories and criteria (IUCN 2024). For comparison with the unknown species and related specimens in herbaria, monographs and contributions of Li (1979, 2010). were also referenced.

### ﻿Material sampling and DNA extraction

Samples of the new species were collected from Leishan County, Qiandongnan Miao and Dong Autonomous Prefecture. The plastome sequences of 4 related *Acorus* species (8 accessions) and two outgroup species were obtained from GenBank (http://www.ncbi.nlm.nih.gov). The total genomic DNA was extracted from the fresh leaves using the modified CTAB method (Doyle and Doyle 1987), and libraries were prepared using the TruePrep DNA Library Prep Kit (Vazyme Biotech Co., Ltd, Nanjing, CN). All DNA, samples and vouchers were deposited in the herbarium at Minzu University of China (MUC). Sample information is listed in Suppl. material 1.

### ﻿Plastome sequencing and assembly

Genomic paired-end sequencing was conducted using the Illumina Novaseq 6000 platform. The chloroplast genome was assembled and analyzed using the program NOVOPlasty v. 4.3.1 (Dierckxsens et al. 2017). Annotation was performed with CPGView to determine the initial location of the chloroplast genome and the inverted repeat (IR) region (Liu et al. 2023), with the chloroplast genome of *A.calamus* (NC 054331) serving as a reference. The annotations were manually checked for errors using Zhou et al. (2021) as a reference. The final chloroplast genome of the new species was deposited in the NCBI GenBank under accession numbers PQ456444 and PQ456445.

### ﻿Phylogenetic reconstruction

Fifty-five single-copy protein-coding genes (PCGs) were extracted from 10 chloroplast sequences using the PhyloSuite v. 1.2.3 software (Zhang et al. 2020a; Xiang et al. 2023). These genes were aligned using the MAFFT v. 7.149b algorithm (Katoh et al. 2019). All individual gene alignments were concatenated to create a dataset for phylogenetic analyses. The best-fit model was determined using the Akaike information criterion (AIC) in the ModelFinder program (Kalyaanamoorthy et al. 2017). A maximum likelihood (ML) tree was constructed to ascertain the phylogenetic position of the species, employing IQ-TREE v. 1.6.10. At the same time, Bayesian inference (BI) analysis was performed with MrBayes based on 55 PCGs of three additional *Acorus* species, also through PhyloSuite v. 1.2.3 software. The resulting phylogenetic trees were visualized and rooted with *Colocasiaesculenta* and *Otteliacordata* using iTOL v. 5 for editing (Ivica and Peer 2021).

## ﻿Results

### ﻿Phylogenetic and morphological analysis

**Figure 1.**
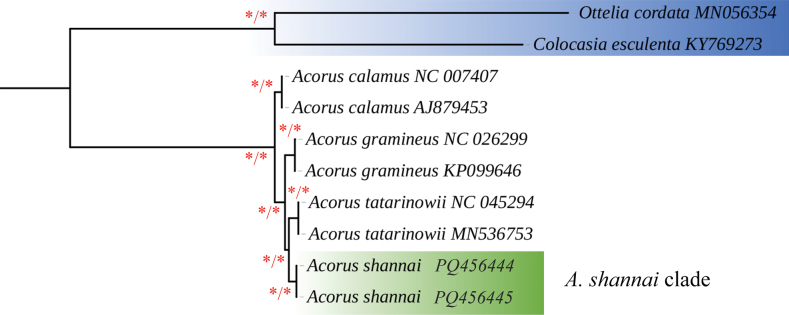
Consensus phylogenetic tree reconstructed by ML and BI analysis based on 55 protein-coding sequences (CDS) of 10 species, with *Colocasiaesculenta* and *Otteliacordata* as outgroups. Asterisks near the branches indicate bootstrap support (BS) percentages obtained from maximum likelihood inference and posterior probabilities (PP) obtained from Bayesian analysis (BS/PP). Those nodes with BS = 100% and PP = 1.00 were shown with asterisks. *A.calamus*NC007407 (Goremykin et al. 2005), *A.calamus*AJ879453, *A.gramineus*NC026299 (Zhu et al. 2016), *A.gramineus*KP099646, “*A.tatarinowii*” NC 045294 (Ma et al. 2020), “*A.tatarinowii*” MN 536753 (Gong et al. 2019), *C.esculenta*KY769273 (Hu et al. 2019), *O.cordata*MN056354 (Zhang et al. 2020b).

The consensus phylogenetic tree, reconstructed by Maximum Likelihood (ML) and Bayesian Inference (BI) analysis based on 55 PCGs from four species of *Acorus*, with *Colocasiaesculenta* and *Otteliacordata* as outgroups, is represented in Fig. 1. The topologies of the ML and BI trees were identical, with all the branches exhibiting strong support (ML BS = 100% and BI PP = 1). All the accessions of *Acorus* formed a monophyletic group with high support. Notably, two samples of the newly described species (*A.shannai* C.L.Long & Z.Cheng, sp. nov.) clustered with a single clade, positioned as sister to the clades of “*A.tatarinowii*” and *A.gramineus* (Fig. 1).

### ﻿Taxonomic treatment

#### ﻿ Acorus shannai

Taxon classificationPlantaeAcoralesAcoraceae

C.L.Long & Z.Cheng sp. nov.

C2A34EBE-E700-5F18-812E-08D6F9B48F4F

urn:lsid:ipni.org:names:77355471-1

**Figure 2.**
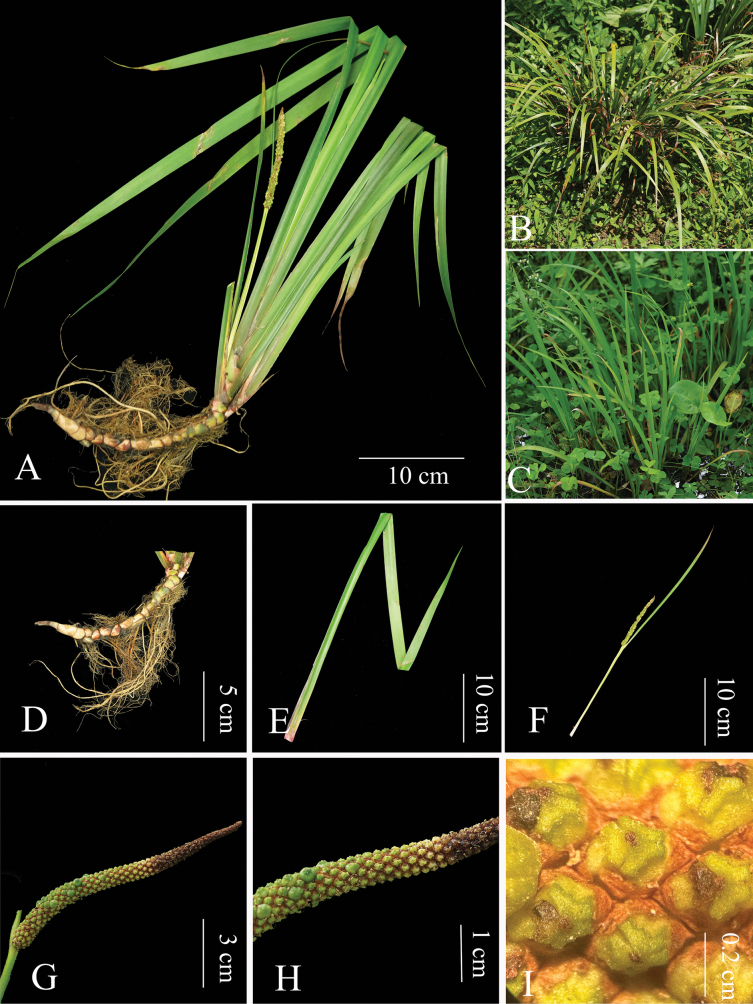
*Acorusshannai* C.L.Long & Z.Cheng, sp. nov. **A** plant inflorescence stage **B** whole plant **C** species habitat **D** rhizome and fibrous roots **E** petiole leaf **F** inflorescence and bract **G, H** inflorescence **I** fruit—photos by Zhuo Cheng.

**Figure 3.**
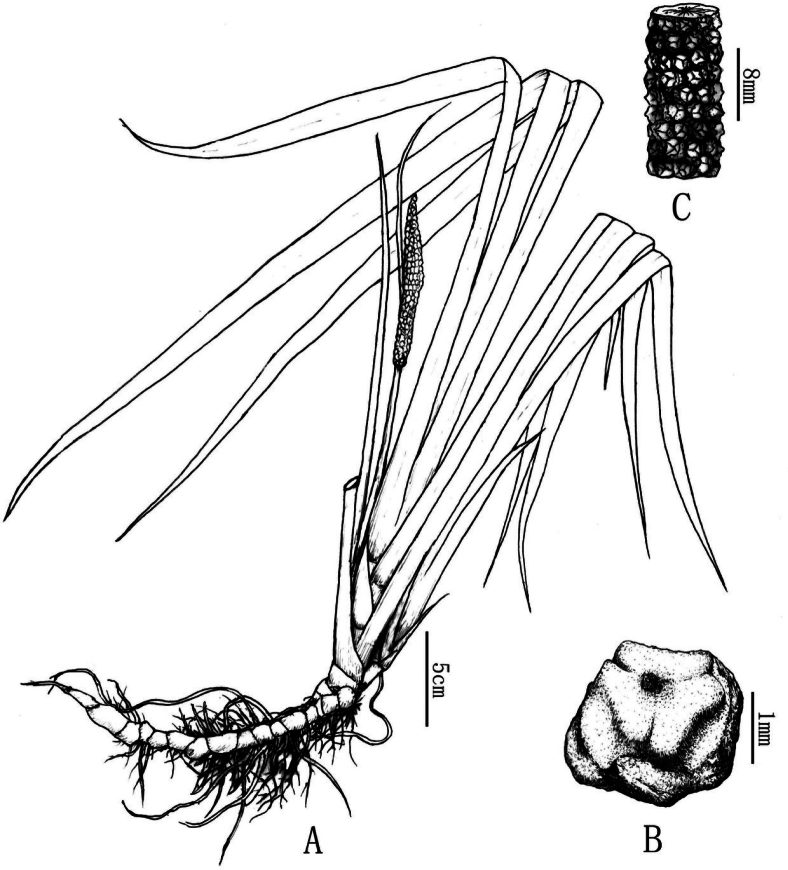
*Acorusshannai* C.L.Long & Z.Cheng, sp. nov. **A** whole plant in infructescence stage **B** fruit **C** infructescence Drawn by Xinchen Qu.

Figs 2 , 3 “山柰菖蒲”(Shan Nai Chang Pu)

##### Diagnosis.

The strong fennel aroma of the plant, distinct dorsal sutures on the fruit, and a spathe length 2–3 times that of the spadix are diagnostic features that differentiate *A.shannai* from other species in the *A.gramineus* group. The new species belongs to the *A.gramineus* group based on the following characteristics: Leaves without conspicuous marginal swellings of the lamina and a distinct midrib (Li 1979). According to the key to the species of *Acorus* occurring in China (Li 1979; Li et al. 2010), the morphology of *A.shannai* is similar to “*A.tatarinowii*” and *A.gramineus*. However, *A.shannai* can be clearly distinguished by the following features: the whole plant emits a strong fennel odor (*vs.* aromatic in “*A.tatarinowii*” and *A.gramineus*), the fruit has distinct dorsal sutures (*vs.* inconspicuous dorsal sutures in “*A.tatarinowii*” and *A.gramineus*), and the leaf-shaped spathe is approximately 2–3 times longer than the spadix (*vs.* more than 3 times in “*A.tatarinowii*”).

##### Type.

China • Guizhou Province: Qiandongnan Miao and Dong Autonomous Prefecture, Leishan County, 26°22'46.5"N, 108°7'53.4"E, alt. 1039 m a.s.l., 25 August 2021; *Zhuo Cheng HXCP024* (KUN!). (holotype: KUN!; isotype: KUN!).

##### Description.

Herbs, perennials, helophyte plants. Plants 40–80 cm, without indumentum. Rhizome recumbent and stout, 10–20 × 0.5–0.8(-1.2) cm, strong *Foeniculumvulgare*–like aroma with fleshy fibrous roots, internodes, 0.6–0.7 cm. Leaf sheath, 0.7–1.5 cm, purple. Leaves several, about ten, light green, ensiform, 30–60 × 0.7–1.5 cm, midrib lacking, apex acuminate. The leaf base is folded in half, the middle is flat above, without distinct midrib, parallel veins up to 15 cm, and exceedingly slender but raised. Peduncles compressed triangular, 8–25 cm. Spadix is green and leaf-like, 10–41 cm., 2–3 times longer than Spadix. Spadix is straight or slightly curved, narrowly cylindrical to subcylindric, 5–13 × 0.5–0.7 cm, densely flowered, 3–4 flowers in one cross-section. Flowers are white, 1.5–1.7 mm. Infructescence, up to 1 cm thick, fruit sub oblong, yellow-green, 4–4.5 × 2–3 mm. Seeds oblong-ellipsoid to ovoid, 2.5–3 × 1–1.2 mm. Flower, May-June. Fruit, July-August. 2n = 24.

##### Etymology.

The specific epithet is derived from the local name “*shannai*”.

##### Distribution, habitat, and phenology.

**Figure 4.**
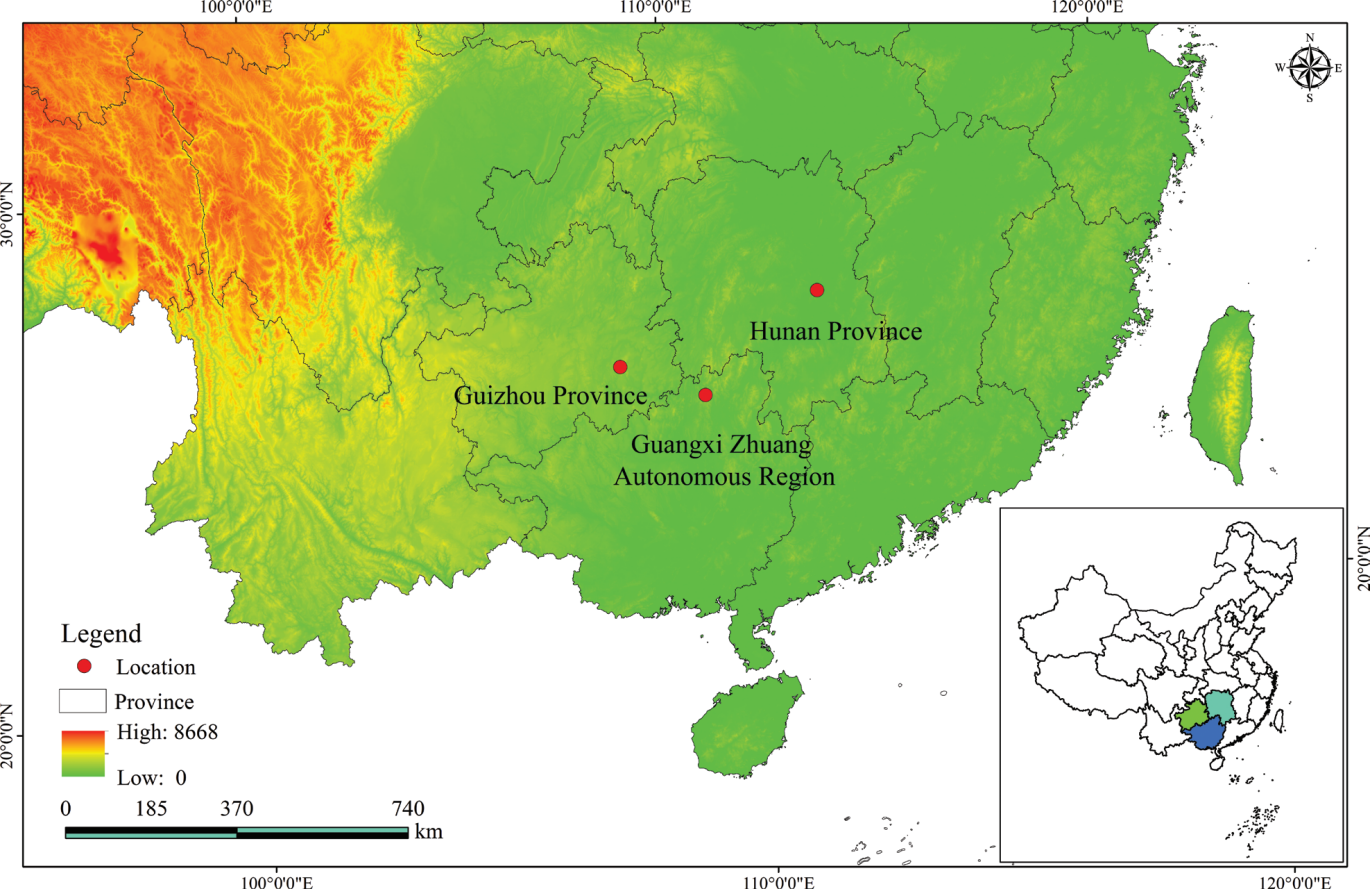
The distribution of *A.shannai* (red circle) in China.

The locality of this taxon is Leishan County, located in the Qiandongnan Miao and Dong Autonomous Prefecture of Guizhou Province. Specimen records indicate this species is also distributed in Guangxi, Hunan, and surrounding areas (Fig. 4). *A.shannai* exhibits a preference for moist mountain slopes and brook-adjacent habitats, distinguishing it ecologically from other *Acorus* species. It typically grows at an elevation of less than 1,500 m a.s.l. The species has been observed flowering in May to June and fruiting in July to August.

##### Taxonomic notes.

The new species identified belong to the *A.gramineus* group, characterized by the following characteristics: leaves without conspicuous marginal swellings of the lamina and a distinct midrib (Li 1979). Previous studies have demonstrated the presence of three species within this group in China based on multiple lines of evidence: *Acorusmacrospadiceus*, “*A.tatarinowii*” and *A.gramineus* (Cheng et al. 2020). Through a comparative analysis of morphological characteristics, distribution locations, and type specimens, we have concluded that it is incorrect to elevate A.gramineusvar.macrospadiceus Yamam. to species level.

According to the key to the species of *Acorus* occurring in China (Li 1979; Li et al. 2010), the morphology of *A.shannai* is similar to “*A.tatarinowii*” and *A.gramineus*. However, *A.shannai* can be distinguished by several features: the whole plant has a strong fennel odor, most of the fruits remain immature, and the leaf-shaped spathe is approximately 2–3 times longer than the spadix. A comparative summary of the characters among these three taxa is presented in Table 1.

**Table 1.** Comparative morphological traits of *Acorusshannai*, “*A.tatarinowii*”, and *A.gramineus* highlighting diagnostic features.

Characters	*A.shannai*	“*A.tatarinowii*”	*A.gramineus*
Whole plant	Leaves drooping	Leaves upright	Leaves upright
Rhizome	Dark green, 8–20 × 0.7–1.2 cm.	Green, 5–15 × 0.5–1 cm.	Dark green, 3–8 × 0.4–0.6 cm.
Leaf blade	Wide, 30–60 × 0.7–1.5 cm	Wide, 20–50 × 0.7–1.3 cm	Narrow, 20–30 × < 0.7 cm
Sheath	Purple	Green	Green
Peduncle	8–25 cm	4–15 cm	2.5–9 (15) cm
Spadix	5–13 cm	(2.5) 4–6.5 (8.5) cm	3–9.5 cm
Spathe	10–41 cm	13–25 cm	3–9 (14) cm
The length ratio of spadix vs spathe	ca. 2–3 times	More than 3 times	ca. 1–3 times
Infructescence	Yellow	Green	Green
Fruit	Most of the fruits kept immature, fruit with distinct dorsal sutures	Most of the fruits were mature, fruit without distinct dorsal sutures	Most of the fruits were mature, fruit without distinct dorsal sutures
Smell	Strong fennel aroma	Aromatic	Aromatic
Habitat	Growing on mountain slopes with moist soil or brook banks; < 1,500 m	Growing on rocks or rocky banks of brooks with fast-flowing water; alt. < 2,600 m	Growing in forests or on stream banks; < 1,800 m

From a geographical distribution perspective, “*A.tatarinowii*” and *A.gramineus* are distributed in southern China, whereas *A.shannai* is limited to a few provinces, with east Guizhou serving as its distribution center. The ecological habits of these species differ significantly. “*A.tatarinowii*” is an aquatic plant, while *A.gramineus* and *A.shannai* are terrestrial taxa. “*A.tatarinowii*” is an epiphytic plant on rocks and in fast-flowing brooks. *A.gramineus* grows in drier soil with lower humidity, and *A.macrospadiceus* grows in moist soil with higher humidity (Cheng et al. 2020).

Our previous research demonstrated that the volatile oil extracted from both the rhizomes and leaves of *A.macrospadiceus* primarily contains estragole, *β*-caryophyllene, trans-anethole, *β*-elemene, and endo-borneol, and estragole is the main component (Shu et al. 2018b). In contrast, it has been reported that the main volatile components of “*A.tatarinowii*” were α-asarone, *β*-asarone, and γ-asarone through GC-MS analysis, with *β*-asarone being presented in the highest quantities (Jaiswal et al. 2015).

In terms of traditional uses, “*A.tatarinowii*” has widely been used as herbal medicine for multiple medicinal purposes in China for more than 2,000 years, particularly for treating central nervous system diseases. It has been included in the Chinese Pharmacopoeia since its first edition as a foundational plant in traditional Chinese medicine known as *Shichangpu*. *A.gramineus* is often used in creating miniascapes because of its unique aroma and ornamental value. Meanwhile, *Acorusshannai* serves as a common seasoning in dishes featuring Artiodactyla or fish in southern China, especially in markets of Guizhou and Guangxi, southern Yunnan, western Hubei and Hunan. Local ethnic groups and Han Chinese have also removed wild populations of *A.shannai* to their gardens for easier access.

The local herbal traders often purchase and sell *A.shannai* in large quantities, mistakenly labeling it as “*A.tatarinowii*”, which may lead to overharvesting. More importantly, due to the varying chemical compositions among different species, the misuse or abuse of the wrong *Acorus* species could pose a threat to clinical safety.

The formal recognition of *A.shannai* contributes to the resolution of long-standing ambiguities within the *A.gramineus* group and highlights the importance of integrating morphological and genomic approaches in plant taxonomy.

##### Preliminary conservation status.

*Acorusshannai* is commonly used as a seasoning in southern China, particularly in regions such as Guizhou, Chongqing, Guangxi, and the surrounding areas. It is commonly found in local markets and is favored for its aromatic properties in culinary applications, especially in meat and fish dishes. However, numerous specimens recorded of this plant in various herbariums indicate a significant interest in its botanical classification. Research indicates that most *A.shannai* resources are derived from cultivation rather than wild populations, which appear to be limited. Without comprehensive biogeographical studies, A.shannai may be classified as “data deficient” (DD) according to IUCN criteria. This classification arises from insufficient information to assess its risk of extinction based on distribution and population status (IUCN 2024). Moreover, the unclear taxonomic status of *A.shannai* leads to confusion in the marketplace, where it is often sold under the name “*A.tatarinowii*”. This misidentification poses risks not only to drug safety but also threatens the sustainable use of *A.shannai* populations. The distinction between these two species is crucial as they are perceived differently by local communities; while they share morphological similarities, locals differentiate them based on their aroma and habitat preferences. Given the reliance on cultivated populations for culinary use and the limited distribution of wild populations, targeted conservation strategies, such as habitat protection and propagation, are critical for sustainable utilization.

##### Additional *A.shannai* specimens examined (paratypes).

*Acorusshannai* – China • Guizhou: Qiandongnan Miao and Dong Autonomous Prefecture, Leishan County, 26°22'46.5"N, 108°7'53.4"E, 1039 m a.s.l., 25 August 2021, Zhuo Cheng HXCP031 (KUN, 5 duplicates) • Guangxi: Longsheng County, 21 March 2014, Longsheng County census team, 450328140321050LY (IBK) • Longsheng County, 10 April 1984, F.N. Wei 01713133 (PE) • Jinxiu Yao Autonomous County, 9 June 1958, Y.K. Li 00141407 (IBK) • Guangdong: Fengshun County, 19 May 1958, X.G. Li 00141420 (IBK) • Hunan: Nanyue District, 10 April 1944, B.H. Liang 00141422 (IBK) • Chengbu County, 16 May 2015, D.G. Zhang et al. CB20150516_GT0151 (JIU).

### ﻿Key to the species of *Acorus* in China

There are four species of *Acorus* distributed in China. An identification key is presented below:

**Table:** 

1	Leaves with distinct midrib, 90 (-150) × 1–2 (-3) cm	***Acoruscalamus* s.l.**
–	Leaves without distinct midrib	**2**
2	Whole plants have a strong fennel odor, and most fruits were not mature	***Acorusshannai***
–	Whole plants do not have a strong fennel odor, and most of the fruits were mature	**3**
3	Spathe short, 3–9 (14) cm, approximately than spadix 1–3 times; leaves narrow, < 0.7 cm	***Acorusgramineus***
–	Spathe long, 13–25 cm, approximately longer than spadix 3 times; leaves wide, > 0.7 cm	“ ***Acorustatarinowii*** ”

## Supplementary Material

XML Treatment for Acorus shannai

## ﻿Additional information

### Author ORCIDs

Zhuo Cheng https://orcid.org/0000-0001-7807-2571

Xinyu Liu https://orcid.org/0009-0007-6299-1283

Nianting Yu https://orcid.org/0009-0005-9469-6567

Weizhe Chen https://orcid.org/0009-0009-2600-9146

Haofeng Zhao https://orcid.org/0009-0009-9597-0600

Feifei Li https://orcid.org/0000-0003-4733-6268

Chunlin Long https://orcid.org/0000-0002-6573-6049

### Data availability

All of the data that support the findings of this study are available in the main text or Supplementary Information.

## Supplementary materials

Supplementary material 1 Accession number of 10 sequenced or downloaded chloroplast genome in this study Data type xlsx https://binary.pensoft.net/file/1224011This dataset is made available under the Open Database License (http://opendatacommons.org/licenses/odbl/1.0/). The Open Database License (ODbL) is a license agreement intended to allow users to freely share, modify, and use this Dataset while maintaining this same freedom for others, provided that the original source and author(s) are credited.Author: Zhuo Cheng

## References

[B1] Cheng Z Shu H Zhang S Luo BS Gu RH Zhang RF Ji YY Li FF Long CL (2020) From folk taxonomy to species confirmation of *Acorus* (Acoraceae): Evidences based on phylogenetic and metabolomic analyses. Frontiers in Plant Science 11: 965. 10.3389/fpls.2020.00965 PMC7327505 32670345

[B2] Dierckxsens N Mardulyn P Smits G (2017) NOVOPlasty: De novo assembly of organelle genomes from whole genome data. Nucleic Acids Research 45: e18. 10.1093/nar/gkw955 PMC5389512 28204566

[B3] Doyle JJ Doyle JL (1987) A rapid DNA isolation procedure for small quantities of fresh leaf tissue. Phytochemical Bulletin 19: 11–15.

[B4] Gong YX Tian YH Nan J Yu WB (2019) Complete plastome sequence of *Acorustatarinowii* (Acoraceae), a traditional Chinese medicinal plant from Xishuangbanna, Yunnan, China. Mitochondrial DNA. Part B, Resources 5(1): 226–228. 10.1080/23802359.2019.1694852 PMC7720961 33366498

[B5] Goremykin VV Holland B Hirsch-Ernst KI Hellwig FH (2005) Analysis of *Acoruscalamus* chloroplast genome and its phylogenetic implications. Molecular Biology and Evolution 22(9): 1813–1822. 10.1093/molbev/msi173 15930156

[B6] Hu H Liu JG Wang BL An JX Wang Q (2019) Characterization of the complete chloroplast genome of *Amorphophalluskonjac* (Araceae) and its phylogenetic analysis. Mitochondrial DNA. Part B, Resources 4(1): 1658–1659. 10.1080/23802359.2019.1606683

[B7] IUCN (2024) Guidelines for Using the IUCN Red List Categories and Criteria. Version 16. Prepared by the Standards and Petitions Committee. https://www.iucnredlist.org/resources/redlistguidelines [Accessed 9^th^ November 2024]

[B8] Ivica L Peer B (2021) Interactive Tree Of Life (iTOL) v5: An online tool for phylogenetic tree display and annotation. Nucleic Acids Research 49(W1): W293–W296. 10.1093/nar/gkab301 PMC8265157 33885785

[B9] Jaiswal Y Liang Z Ho A Chen H Zhao Z (2015) Metabolite profiling of tissues of *Acoruscalamus* and *Acorustatarinowii* rhizomes by using LMD, UHPLC-QTOF MS, and GC-MS. Planta Medica 81(4): 333–341. 10.1055/s-0035-1545694 25760385

[B10] Kalyaanamoorthy S Minh BQ Wong TKF Haeseler AV Jermiin LS (2017) ModelFinder: Fast model selection for accurate phylogenetic estimates. Nature Methods 14(6): 587–589. 10.1038/nmeth.4285 28481363 PMC5453245

[B11] Katoh K Rozewicki J Yamada KD (2019) MAFFT online service: Multiple sequence alignment, interactive sequence choice and visualization. Briefings in Bioinformatics 20(4): 1160–1166. 10.1093/bib/bbx108 28968734 PMC6781576

[B12] Li H (1979) Araceae. Flora Reipublicae Popularis Sinicae. Science Press, Beijing 13(2): 4–9. [The Chinese edition of Flora of China]

[B13] Li H Zhu GH Bogner J (2010) Acoraceae. In: Wu ZY Raven PH Hong DY (Eds) Flora of China. Science Press, Beijing, and Missouri Botanical Garden Press, St.Louis 23: 1–2.

[B14] Liu SY Ni Y Li JL Zhang XY Yang HY Chen HM Liu C (2023) CPGView: A package for visualizing detailed chloroplast genome structures. Molecular Ecology Resources 23(3): 694–704. 10.1111/1755-0998.13729 36587992

[B15] Ma L Jiang SZ Lian H Xiong YF Liu ZJ Chen SP (2020) The complete chloroplast genome sequence of *Acorustatarinowii* (Araceae) from Fujian, China. Mitochondrial DNA. Part B, Resources 5(3): 3159–3160. 10.1080/23802359.2020.1806133 PMC7782871 33458094

[B16] Shu H Zhang S Lei QY Zhou J Ji YY Luo BS Hong LY Li FF Liu B Long CL (2018a) Ethnobotany of *Acorus* in China. Acta Societatis Botanicorum Poloniae 87(2): 3585. 10.5586/asbp.3585

[B17] Shu H Morcol T Zheng J Zhao L Kennelly EJ Long CL (2018b) Studies on volatile oils compounds of *Acorusmacrospadiceus* based on GC-MS, an ethnomedicinal and food plant used by local people in southwest China. China Journal Chinese Materia Medica 43: 1774–1779. 10.19540/j.cnki.cjcmm.20180119.001 29902885

[B18] Sokoloff DD Sennikov AN (2023) (2996) Proposal to conserve the name Acoruscalamusvar.americanus (*A.americanus*) (Acoraceae) with a conserved type. Taxon 72(6): 1366–1368. 10.1002/tax.13089

[B19] Sokoloff DD Remizowa MV Nuraliev MS Averyanov LV Sennikov AN (2023a) The first genome from the basal monocot family has been misnamed: Taxonomic identity of *Acorustatarinowii* (Acoraceae), a source of numerous chemical compounds of pharmaceutical importance. Diversity 15(2): 176. 10.3390/d15020176

[B20] Sokoloff DD Remizowa MV Severova EE Sennikov AN (2023b) Inference of ploidy level in 19^th^-Century historical herbarium specimens reveals the identity of five *Acorus* species described by Schott. Diversity 15(6): 766. 10.3390/d15060766

[B21] Sokoloff DD Remizowa MV Skaptsov MV Yadav SR Sennikov AN (2023c) Back to Linnaeus: Proper botanical naming of the tetraploid Indian *Acorus* (Acoraceae), an important medicinal plant. Diversity 15(6): 785. 10.3390/d15060785

[B22] Sokoloff DD Degtjareva GV Skaptsov MV Vislobokov NA Kirejtshuk AG Sennikov AN Severova EE Chepinoga VV Samigullin TH Valiejo-Roman CM Smirnov SV Shmakov AI Marchuk EA Remizowa MV (2024) Diploids and tetraploids of *Acorus* (Acoraceae) in temperate Asia are pseudocryptic species with clear differences in micromorphology, DNA sequences and distribution patterns, but shared pollination biology. Taxon 73(3): 718–761. 10.1002/tax.13173

[B23] Sokoloff DD Degtjareva GV Skaptsov MV Vislobokov NA Kirejtshuk AG Sennikov AN Severova EE Chepinoga VV Samigullin TH Valiejo-Roman CM Smirnov SV Shmakov AI Marchuk EA Remizowa MV (2024a) Diploids and tetraploids of *Acorus* (Acoraceae) in temperate Asia are pseudocryptic species with clear differences in micromorphology, DNA sequences and distribution patterns, but shared pollination biology. Taxon 73(3): 718–761. 10.1002/tax.13173

[B24] Sokoloff DD Degtjareva GV Valiejo-Roman CM Severova EE Barinova S Chepinoga VV Kuzmin IV Sennikov AN Shmakov AI Skaptsov MV Smirnov SV Remizowa MV (2024b) Kazakhstan has an unexpected diversity of medicinal plants of the genus *Acorus* (Acoraceae) and could be a cradle of the triploid species *A.calamus*. Plants 13(14): 1978. 10.3390/plants13141978 PMC11281264 39065505

[B25] Sun ZC Wang ZG Qu F Su XK Lin YC Yan H Long W Zhu GF Zhao TM (2024) Integrated valorization of *Acorusmacrospadiceus* by comprehensive evaluation of hydro-distilled essential oil and residual non-volatile extracts. Waste and Biomass Valorization, 1–12. 10.1007/s12649-024-02742-7

[B26] Thiers B (2020) [continuously updated] Index Herbariorum. A global directory of public herbaria and associated staff. New York Botanical Garden’s Virtual Herbarium. http://sweetgum.nybg.org/science/ih [Accessed 9^th^ November 2024]

[B27] Wang ZB Wang QH Yang BY Li J Yang CJ Meng YH Kuang HX (2014) GC-MS method for determination and pharmacokinetic study of four phenylpropanoids in rat plasma after oral administration of the essential oil of *Acorustatarinowii* Schott rhizomes. Journal of Ethnopharmacology 155(2): 1134–1140. 10.1016/j.jep.2014.06.035 25046827

[B28] Wei FN Li YK (1985) A new spice, *Acorusmacrospadiceus* from south China. Guihaia 5(3): 179–182.

[B29] Xiang CY Gao FL Jakovlic I Lei GP Hu Y Zhang H Zou H Wang GT Zhang D (2023) Using PhyloSuite for molecular phylogeny and tree - based analyses. iMeta 2(1): e87. 10.1002/imt2.87 PMC10989932 38868339

[B30] Yamamoto Y (1943) Contributiones ad floram Kainanensem (΄Kainanensis΄) I. Kainan Kaigun Tokumubu Seimukyoku, Taihoku, Japan.

[B31] Zhang D Gao FL Jakovlic I Zou H Zhang J Li WX Wang GT (2020a) PhyloSuite: An integrated and scalable desktop platform for streamlined molecular sequence data management and evolutionary phylogenetics studies. Molecular Ecology Resources 20(1): 348–355. 10.1111/1755-0998.13096 31599058

[B32] Zhang QF Shen ZX Li FY Li GJ Shen J (2020b) Complete chloroplast genome sequence of an endangered *Otteliacordata* and its phylogenetic analysis. Mitochondrial DNA. Part B, Resources 5(3): 2209–2210. 10.1080/23802359.2020.1768921 PMC7510642 33366975

[B33] Zhou F Lan KK Li XR Mei Y Cai SK Wang JH (2021) The complete chloroplast genome sequence of *Vernoniaamygdalina* delile. Mitochondrial DNA. Part B, Resources 6(3): 1134–1135. 10.1080/23802359.2021.1902411 PMC7995831 33796766

[B34] Zhu A Guo W Gupta S Fan W Mower JP (2016) Evolutionary dynamics of the plastid inverted repeat: The effects of expansion, contraction, and loss on substitution rates. The New Phytologist 209(4): 1747–1756. 10.1111/nph.13743 26574731

